# On the Analyses of Cure Cycle Effects on Peel Strength Characteristics in Carbon High-*T_g_* Epoxy/Plasma-Activated Carbon PEEK Composite Interfaces: A Preliminary Inquiry

**DOI:** 10.3390/ma16237340

**Published:** 2023-11-25

**Authors:** Henri Perrin, Régis Vaudemont, Masoud Bodaghi

**Affiliations:** Luxembourg Institute of Science and Technology (LIST), 5 rue Bommel, L-4940 Hautcharage, Luxembourg; henri.perrin@list.lu (H.P.); regis.vaudemont@list.lu (R.V.)

**Keywords:** co-curing bonding, plasma treatment, PEEK/epoxy interphase

## Abstract

In this study, a high-*T_g_* aerospace-grade epoxy composite plate was co-curing welded using a unidirectional PEEK thermoplastic carbon fibre tape to develop advanced composite joints. To account for the surface roughness and the weldability of carbon–epoxy/carbon–PEEK composites, plasma treatments were performed. The co-curing was conducted by the following steps: each treated thermoplastic tape was first placed in the mould, and followed by nine layers of dry-woven carbon fabrics. The mould was sealed using a vacuum bag, and a bi-component thermoset (RTM6) impregnated the preform. To understand the role of curing kinetics, post-curing, curing temperature, and dwell time on the quality of joints, five cure cycles were programmed. The strengths of the welded joints were investigated via the interlayer peeling test. Furthermore, cross-sections of welded zones were assessed using scanning electron microscopy in terms of the morphology of the PEEK/epoxy interphase after co-curing. The preliminary results showed that the cure cycle is an important controlling parameter for crack propagation. A noticeable distinction was evident between the samples cured first at 140 °C for 2 h and then at 180 °C for 2 h, and those cured initially at 150 °C for 2 h followed by 180 °C for 2 h. In other words, the samples subjected to the latter curing conditions exhibited consistently reproducible results with minimal errors compared to different samples. The reduced errors confirmed the reproducibility of these samples, indicating that the adhesion between CF/PEEK and CF/RTM6 tends to be more stable in this curing scenario.

## 1. Introduction

Composite materials, both thermoplastic and thermoset, have found extensive applications in land transportation, aerospace, and marine structures, gradually replacing their traditional metallic counterparts [1,2]. This shift is primarily attributed to the superior strength and stiffness-to-weight ratios offered by composite materials when compared to metals. The superior specific properties of composites, in contrast to metals, result in reduced weight, increased payload capacity, extended operational range, and improved overall mechanical performance of structures.

There has been enormous attention from researchers and the aerospace industry alike to the use of thermoplastic composites (TPCs). Advanced thermoplastic composites present numerous processing and mechanical performance benefits compared to thermoset composites. These advantages include high damage tolerance, exceptional corrosion and solvent resistance, elevated fracture toughness, superior impact resistance, commendable fatigue resistance, economical storage costs, and an indefinite shelf life [2]. An example of this is the thousands of press-formed carbon fibre/polyetheretherketone (CF/PEEK) clips that were previously built into the A350 and Boeing 787 aircraft [3].

Compared to their thermoplastic counterparts, the jointing and assembly of CF/Epoxy composites pose a significant challenge for producing large-scale complex composite structural parts. Due to the high level of cross-linking, fusion bonding is no longer viable for cured CF/epoxy composites [2,4,5,6,7]. Instead, conventional methods such as mechanical fastening and adhesive bonding are employed as alternative solutions. The CF/PEEK is now attached to the CF/epoxy fuselage skin via mechanical fastening, which is a well-established technique for aircraft metallic structures [8]. However, these methods have their own inherent drawbacks when it comes to joining and assembling composite parts. With mechanical fastening, challenges typically arise from stress concentrations, potential delamination induced by drilling, potential galvanic corrosion, limitations in lightweight design, and the time-consuming, labor-intensive nature of the process [9,10,11]. In the case of adhesive bonding, extensive surface preparation and long curing cycles are necessary, making it inherently incompatible with the requirements of mass production [4,11,12].

Fusion bonding is considered an optimal technique for joining thermoplastic matrix composites. It involves three key stages: first, heating the thermoplastic matrices at the interface to achieve a melted state; next, applying pressure to the paired parts to facilitate the inter-diffusion of polymer chains; and finally, allowing the composite to cool down and regain its mechanical properties [13]. A relevant question is how the existing techniques can be applied for the welding of dissimilar composites, i.e., TPC and thermoset composite (TSC) combinations.

To expand the use of fusion bonding in thermoset polymer matrix composites, it is necessary to coat the interface of the laminates with a layer of thermoplastic material [14]. However, the thermoplastic hybrid interlayer method may encounter challenges in achieving the desired through-thickness inter-penetration depth of the woven roving fiber cloth. This difference in melt viscosity can be attributed to the significant contrast between the thermoplastic film and the thermoset matrix resin. Moreover, the elevated temperature needed to melt the thermoplastic hybrid interlayer or film may result in the degradation of the thermoset matrix resin. One of the most common solutions to increase the occurrence of interfacial adhesion is using a thermoplastic interlayer that is compatible with the thermoset resin in TSC [9]. Some examples of such interlayer thermoplastic resins are polyetherimide (PEI), polysulfone (PSU), and polyethersulfone (PES), which are of an amorphous nature [14]. In addition to the compatibility of the inter-layer thermoplastic resins with the thermoset matrix, a few other factors must also be considered to select the appropriate inter-layer thermoplastic resin: mechanical and environmental performance in applications, techniques used to incorporate the interlayers, and weldability of the interlayer thermoplastic resin without thermoset degradation. To achieve a sufficient high-strength bond without influencing the thermoset cure cycle, the chemical and physical properties of the interlayer and thermoset must also be considered. Moreover, issues such as resistance to aircraft solvents and moisture must also be addressed [14]. Consequently, it typically requires intermediate pretreatment involving plasma and corona discharge to enhance its interfacial adhesion with the thermoset matrix. One significant advantage of plasma treatment is that it preserves material integrity, reducing the likelihood of fibre degradation compared to chemical treatment. Additionally, plasma treatment is a clean and dry process, mitigating environmental concerns typically associated with chemical modification [15]. The surface of the TPC can be treated by the atmospheric plasma to generate a certain surface roughness that will cause mechanical interlock between the TSC and TPC during the welding process [16]. This solution does not require the interlayer and makes direct welding of a TPC on the treated surface of TSC possible. Understandably, some of the main points of concern in this solution are the weld strength and durability of the thermoplastic and thermoplastic connection.

This study proposes a new technique to achieve a high-strength connection of the hybrid interface by co-curing the unidirectional carbon fiber-reinforced thermoplastic tape with the thermoset composite without the requirement of an interlayer thermoplastic. This enables us to directly test the interface using the peeling test without an additional welding process, like infrared welding, which could be responsible for thermal degradation of the TSC substrate.

Co-curing, a method for joining polymer composite joints, involves simultaneous curing, offering cost-effectiveness compared to co-bonding or secondary bonding techniques [17,18,19]. Co-bonding implies curing stacked prepregs alongside other parts, while secondary bonding involves bonding cured parts using adhesive. After establishing the concept, the surface of the CF/epoxy composite laminates underwent a coating process using various thermoplastic binders in either powder or film form. Subsequently, the composites were co-cured through hot pressing. It was anticipated that the elevated curing temperature would induce the melting of the thermoplastic binder, allowing it to blend with the epoxide resin in the CF/epoxy prepreg under vacuum and/or external pressure. This blend was then consolidated through the curing of the epoxide resin and the thermoplastic binder. As for the heating element, a carbon-fiber-reinforced binder (CF/binder) prepreg interlayer, comprising a single layer of carbon fabric sandwiched between two layers of binder film, was prepared through hot pressing [20].

Several studies have examined thermoset-to-thermoset composite joints. Moretti et al. [21] investigated process-induced strains during autoclave co-curing, co-bonding, and secondary bonding of epoxy composite laminates, noting minimal warpage in the co-curing process. Hasan et al. [22] produced full-scale wing demos joined by co-curing or secondary bonding, revealing significantly less laminate warpage in co-curing bonded joints compared to secondary bonded ones. Furthermore, co-curing bonded demos satisfied engineering tolerances without any defects or anomalies. In addition to its manufacturing quality and accuracy, several studies reported adequate or excellent mechanical properties of co-curing bonded joints [23,24,25]. For instance, Dhilipkumar and Rajesh [25] observed 67% and 52% higher lap shear strengths in co-curing bonded joints compared to co-bonding joints and secondary bonding joints, respectively. Kim et al. [24] reported much higher pull-off strengths in co-curing hat-stiffened panels than those manufactured by co-bonding and secondary bonding techniques. Based on these findings, co-curing emerges as a promising technique for composite joining, offering good structural integrity with minimal curing cycles.

Although there is growing understanding regarding the co-curing of thermoset-to-thermoset composite bonding, the application of this method for joining carbon fiber/PEEK tapes and aerospace carbon fiber/epoxy composites through a co-curing process without an interlayer remains limited. Quan et al. [26] offer insights into the creation of composite joints featuring robust structural integrity and thermal stability through the co-curing bonding of CF/epoxy composites and CF/PEEK tapes. Employing a UV-irradiation technique, the researchers treated the surfaces of PEEK films, a method demonstrated to notably amplify their adhesive properties with epoxies [27].

Given the prevalent use of CF/PEEK and CF/epoxy composites in aerospace applications, this paper concentrates on gaining additional insights into the co-curing process of these materials. The study explores CF/PEEK tapes as substitutes for epoxy adhesives in bonding aerospace carbon fibre/epoxy composites via co-curing. Plasma treatment was employed to modify the PEEK film surfaces, and the investigation encompassed peel testing of the composite joints bonded by CF/PEEK tapes.

## 2. Experimental Details

### 2.1. Materials

For the present investigation, a CF/PEEK tape in its amorphous form (PEEK A) was selected, as indicated in Table 1 [28,29,30]. The employed thermoset resin was RTM6-2, a dual-component resin designed for resin transfer moulding and infusion processes and to meet the demands of the aerospace sector (Table 2) [31,32]. The provided reinforcing fiber came from Hexcel, specifically the HexTow AS4C (Table 3), which consists of a continuous twill-woven carbon fabric derived from PAN [33].

### 2.2. Plasma Treatment

Plasma treatments were conducted utilizing an atmospheric pressure reactor based on dielectric barrier discharge (DBD) principles. In essence, plasma was initiated within a DBD setup with a 3 mm gap between two electrodes. Each of these high-voltage aluminium plate electrodes was shielded by a glass plate measuring 3.25 mm in thickness. The plasma discharge was induced through an AC power supply configured to 450 W and 6 kHz, accompanied by a gas mixture of 80% nitrogen (N_2_) and 20% oxygen (O_2_). The upper electrode was moved consistently at a speed of 4 m/min over the lower electrode. The deposition duration was established at 1 min. For those interested in gaining a more comprehensive understanding of surface treatment duration, the publication [16] by the same author is recommended. The plasma treatment process necessitates strict adherence to health and safety considerations to safeguard personnel and maintain regulatory compliance. Chemical exposure poses a significant concern, as reactive gases utilised in the plasma treatment may lead to respiratory issues, requiring proper ventilation and exposure monitoring. High voltages involved in the operation demand thorough training and the implementation of safety interlocks to prevent electric shocks. Personal protective equipment, including gloves and eye protection, is imperative to shield against chemical exposure and potential splashes.

### 2.3. Co-Curing Process

**Figure 1:**
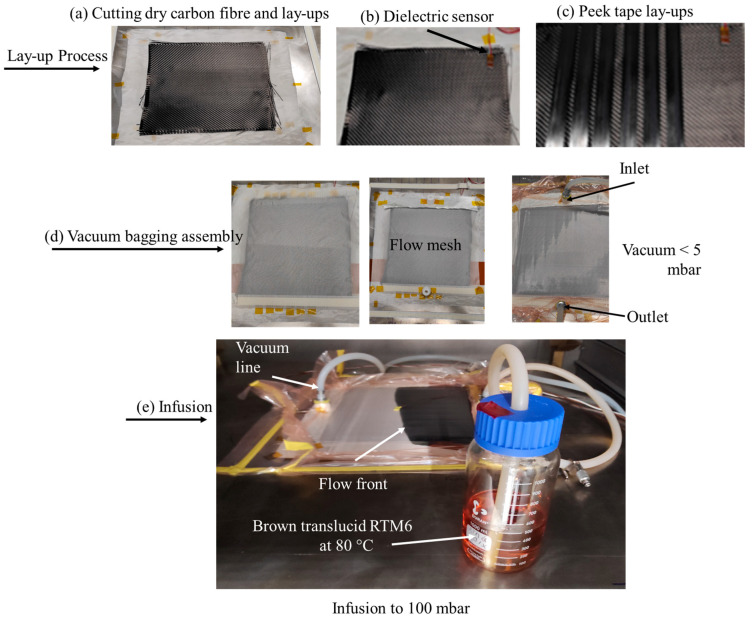
Manufacturing processes.

A descriptive schematic of the co-curing process is shown in Figure 1. Liquid resin infusion (LRI) was utilized for the co-cure processing of the joint interface between CF/PEEK tape and thermoset matrix composites reinforced with woven carbon fibers (Figure 1a). The process started by laying up nine plies of twill-woven carbon fabric from Hexcel (Table 3).

The temperature of the samples was monitored with a dielectric sensor positioned at one end of the preform (Figure 1b). Following this, six CF/PEEK tapes were positioned next to each other on top of the polyimide film (Figure 1c). The CF/PEEK (Aptiv^®^2000) was supplied by Victrex^®^ (Lancashire, UK). The matrix has a glass transition temperature *T_g_* = 143 °C and a recrystallization temperature *T*_C_ = 160 °C. The tapes have an initial fibre volume fraction (V_f_) close to 54 vol% [34]. After placing nine layers of twill-woven carbon fabric on top, the assembly was covered on the upper side with a flow mesh and a flexible bag. A vacuum was applied to remove air from the assembly (Figure 1d). The two-component RTM6-2 [35] was drawn into the assembly at temperatures of 80 °C from the reservoir (Figure 1e). The RTM6 resin is a single-component polyepoxide resin widely used in the aerospace industry. This resin is employed in the manufacturing processes of composites through liquid methods, notably in the LRI process or through injection on reinforcement, such as in resin transfer moulding (RTM).

**Figure 2:**
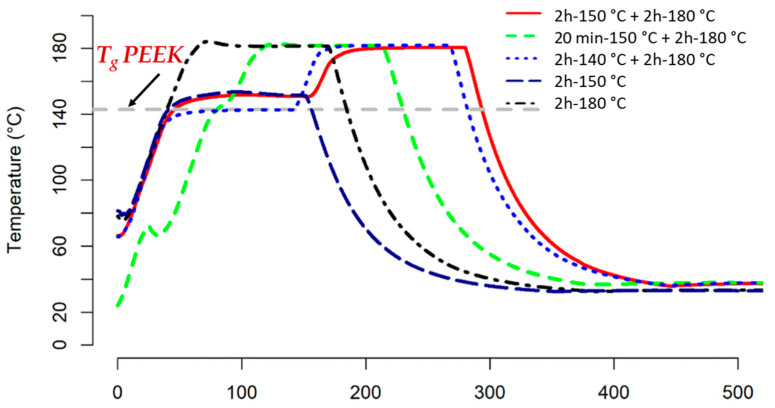
Five different curing cycles.

Following the impregnation process, a total of five distinct cure cycles (Figure 2) were established to investigate the influence of curing cycles on bonding strength. A ramp between 80 °C and the final set temperatures for four distinct cure cycles was 2 °C/min except for the segment 20 min + 150 °C, which was 1.2 °C/min. Then, each temperature segment remained constant for a given time (which varied between 20 min and 2 h) before a cooling ramp at 2 °C/min. For each curing cycle, six (6) samples were tested.

In view of the comparison of TTT diagrams (a time–temperature–transformation diagram obtained) with three sets of parameters from the literature [36,37], we can first observe the similarity in the appearance of the obtained diagrams. However, significant differences exist, especially in terms of time, between the obtained TTT diagrams. This reflects the reality of dispersion in reaction rates as well as the variability in the determination of kinetic parameters. Nevertheless, these graphs provide insight into the behaviour of the resin and assist in predicting the progress achieved by a curing cycle. These graphs highlight the role of vitrification concerning the advancement and reaction kinetics of the resin, with the kinetics being markedly different on either side of vitrification. Most of the literature focused on investigating the reactive characteristics of pure RTM6 has limited practical applicability, as it primarily addresses a commercial system that is never utilized without the reactive binder—a crucial component for achieving the desired properties [38]. Consequently, optimising manufacturing cure cycles was highly valuable. The manufacturing process can be divided into two phases. First, the resin is injected (or drawn) through a dry fabric, known as the impregnation phase. In the second phase, the resin is cured by applying a temperature cycle. Understanding the curing kinetics of a resin is crucial for adjusting the composite curing cycles, not only to match the specific requirements of the part being produced but also to obtain good adhesion between the thermoset and thermoplastic counterparts. Five curing cycles (Figure 2) as a function of temperature and time were, therefore, chosen based on the vitrification curve [39].

### 2.4. Peel Tests

The 90° peel adhesion test according to ILNAS-EN 28510-1:2014 [40] serves the purpose of evaluating the force required to separate the two components that were joined through co-curing. The outcome of this test, akin to bond strength, is denoted as N (force to de-bond)/25.4 mm (tape width). The test was conducted at a speed of 0.84 mm/s and repeated six times. The freely moving table was linked to the crosshead via a pulley and rope mechanism. This arrangement ensured that the table’s lateral movement matches the crosshead’s motion, thereby maintaining a consistent 90° angle between the two components. It is crucial to emphasise that the untreated PEEK/C tape configuration exhibits inadequate adhesion post-demoulding, making peeling tests unviable.

After the peel test, the fracture surfaces were examined using scanning electron microscopy (SEM). The SEM analysis was performed with an FEI Quanta FEG 200 scanning electron microscope from the FEI Company (Hillsboro, OR, USA), operating under pressure-controlled conditions.

## 3. Results and Discussion

### 3.1. Peel Tests

**Figure 3:**
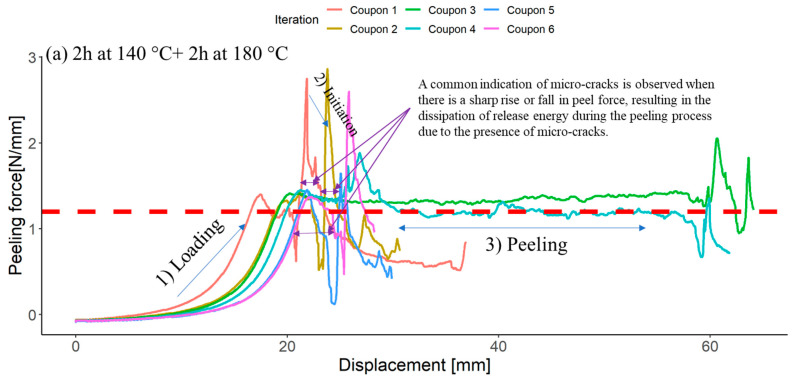

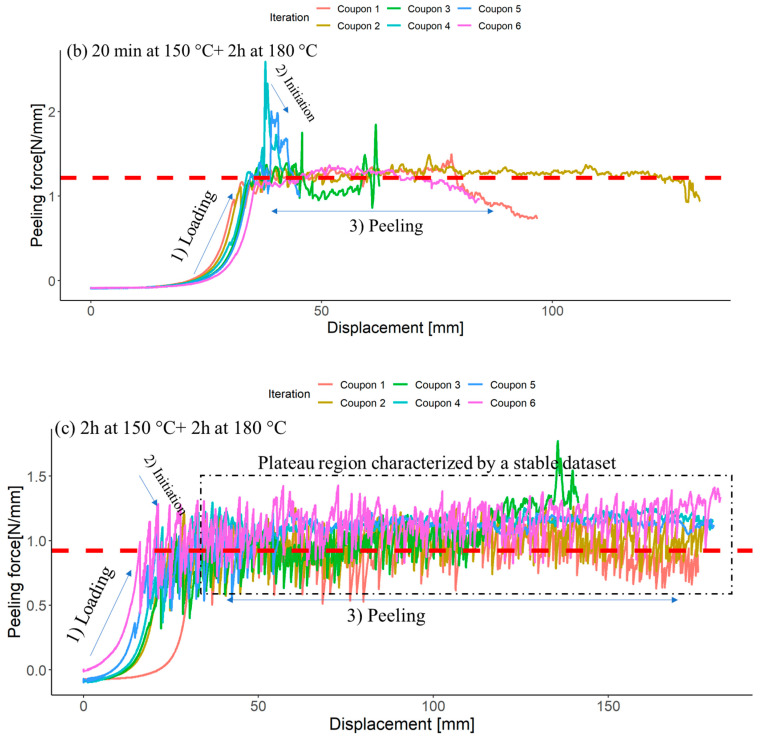

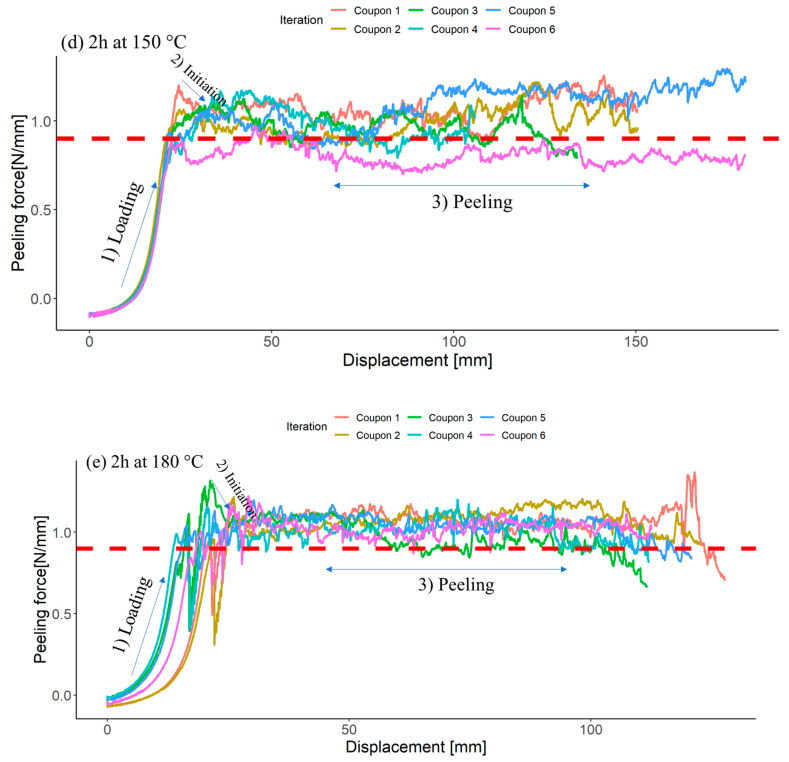
Load displacement for the samples cured at different dwell temperatures and time (six tests per curing cycle): (**a**) 2 h at 140 °C + 2 h at 180 °C, (**b**) 20 min at 150 °C + 2 h at 180 °C, (**c**) 2 h at 150 °C + 2 h at 180 °C, (**d**) 2 h at 150 °C, (**e**) 2 h at 180 °C. The red dashed line represents the computed average peeling force derived from multiple iterations.

Figure 3a–e presents the relationship between peel force and displacement for separately co-cured samples. In general, each curve displays three distinct phases:

1. Initially, there’s an increase in peeling force until the interface starts to propagate.
2. Once the interface begins to propagate, there’s a slight drop in peeling force. As cracks propagate between adhered materials, the effective load transfer diminishes, resulting in a reduction in peeling force. It is important to note that Xu et al. (1992) [41] characterized a viscoelastic plate resembling a cantilever beam subjected solely to bending. Their findings indicate that interfacial toughness initially rises and subsequently declines with an increase in crack propagation velocity. This implies the potential presence of peak viscoelastic energy dissipation occurring at an intermediate crack velocity [42].
3. This is followed by a peeling process where the peeling force stabilises, represented by the average force during peeling (indicated by dashed red lines in Figure 3) in our experiments.

Since the three distinct phases were not easily recognisable, the boundaries of these three phases are indicated by blue arrows for each curing cycle scenario in Figure 3. The force–displacement curves acquired from these tests exhibit diverse patterns contingent upon the cure cycles. Peel arms characterised by heterogeneity generate periodic peaks in the peel curve, exemplified by the peak force, denoted as Fmax in Figure 3a,b. Distinct curve shapes can be attributed to the type or structure of the heterogeneity. In contrast, debond propagation in samples cured at 150 °C for 2 h and at 180 °C for 2 h often displays temporally unstable, or stick–slip crack behaviour—sometimes referred to as “shocky” or “zippy” behaviour, as depicted in Figure 3c. This intricate phenomenon is influenced by multiple factors, including constitutive material properties, test rate, test temperature, specimen and load-train compliance, and system inertia [43]. The associated peel force versus displacement curve in Figure 3c illustrates the intermittent stick–slip growth of the crack. The reasons behind the temporal instability of crack propagation typically involve intricate, multi-physics interactions [44,45], details of which are beyond the scope of this paper.

It is evident from Figure 3 that each curing cycle resulted in a significantly different curve for the peel force versus displacement. The notable difference is apparent between the samples cured initially at 140 °C for 2 h and then at 180 °C for 2 h, and the samples cured initially at 150 °C for 2 h and 180 °C for 2 h. On the other hand, the samples cured initially at 150 °C for 2 h and 180 °C for 2 h showed reproducible results, with the least number of errors among the different samples. Fewer errors confirmed that the samples are reproducible, and the adhesion between CF/PEEK and CF/RTM6 tends to be more stable. The CF/PEEK-RTM6 samples co-cured first at 140 °C for 2 h and then at 180 °C for 2 h (Figure 3a) exhibited significant variability in results without reaching a stable state. This suggests a lack of valid adhesion between PEEK and epoxy.

In the case of co-curing initially at 140 °C for 2 h and subsequently at 180 °C for 2 h, a typical peel result revealed significant force oscillations due to staged crack development, as illustrated in Figure 3a. Figure 3a clearly depicts a notable reduction in peel force [46]. A comparable trend, albeit more moderate, was observed for the co-curing scenario, where the initial step involved curing at 150 °C for 20 min, followed by a subsequent curing at 180 °C for 2 h. This observation emphasises how curing conditions, like temperature and time, impact interphase formation and peel strength. Additionally, the stable crack propagation typically seen in unidirectional (UD) reinforced materials during testing isn’t observed here, as we are dealing with woven fabric composites, particularly tough thermoplastic composites like CF/PEEK [47]. Unlike the previous observation, the results for samples cured at 150 °C for 2 h and 180 °C for 2 h showed a stable dataset. The oscillated crack propagation, especially in samples cured at 150 °C for 2 h and 180 °C for 2 h, is related to collective interactions of contact areas. In this process, when peeling occurs, a crack initiates and moves through the interface faster than the peel arms can move. When the peel arms loosen, the crack stops, and this cycle repeats, creating the characteristic stick–slip pattern [48] seen in Figure 3c. This unstable stick–slip crack growth is associated with structural transitions, such as moving from cohesive to interfacial failure or between different interfacial failure modes, confirming the formation of the PEEK and RTM6 interfaces [49]. Notably, the details of the stick–slip pattern depend on the dwell temperature relative to the glass transition temperature (*T_g_*) of PEEK. Above the *T_g_*, epoxy polymeric chains/PEEK are in a mobile state and can further dissolve/diffuse during the co-curing process, resulting in a thicker interface.

Figure 3d,e also demonstrate that a single-dwell curing cycle may halt the diffusion process, leading to weaker stick–slip behaviour and lower displacement compared to two dwell curing cycles. This effect becomes more pronounced with longer dwell times (2 h) and higher temperatures (150 °C). Specifically, a 2 h dwell at 150 °C and 2 h at 180 °C allow for longer mobility and diffusion times after reaching the nominal gel point. However, when considering the 20 min dwell time, these graphs emphasise the distinct impact of vitrification on the resin’s reaction kinetics. It is evident that the kinetics vary significantly on either side of the vitrification point, depending on the duration. Specifically, at 150 °C, vitrification begins after approximately 100 min [36].

The results of the maximum mean peel force were compared statistically with respect to the curing cycle, as shown in Table 4. The curing cycle clearly exhibited a significant impact. While the peel force for the curing cycle of 2 h at 140 °C + 2 h at 180 °C was the highest compared to other curing cycles, it also showed the highest coefficient of variation, at 22%, confirming a high scatter and oscillation in the peel test results. On the other hand, although the mean peel forces for the samples cured for 2 h at 150 °C + 2 h at 180 °C were more consistent, they still showed a high coefficient of variation compared to the single-dwell curing cycle. This is primarily because one out of six samples showed a sudden increase in peel force due to an earlier failure.

### 3.2. Microscopic Investigation of the Interfaces of the Joints

**Figure 4:**
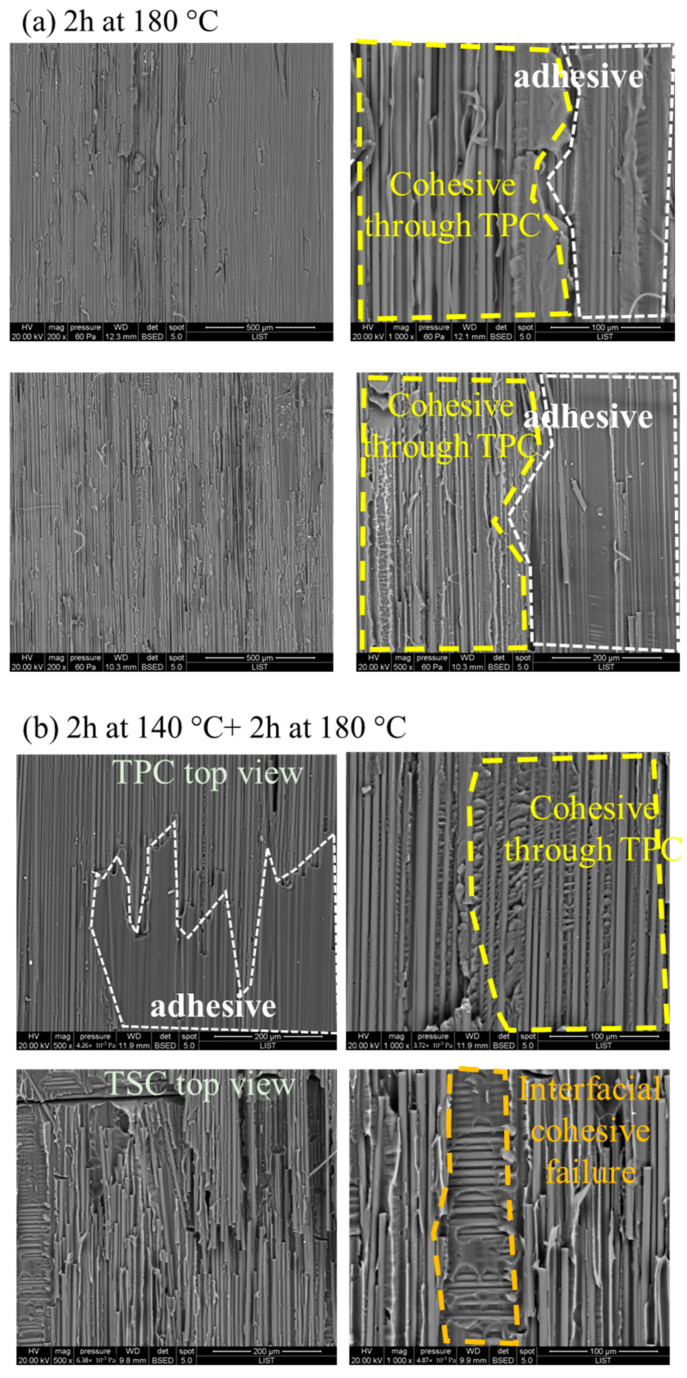

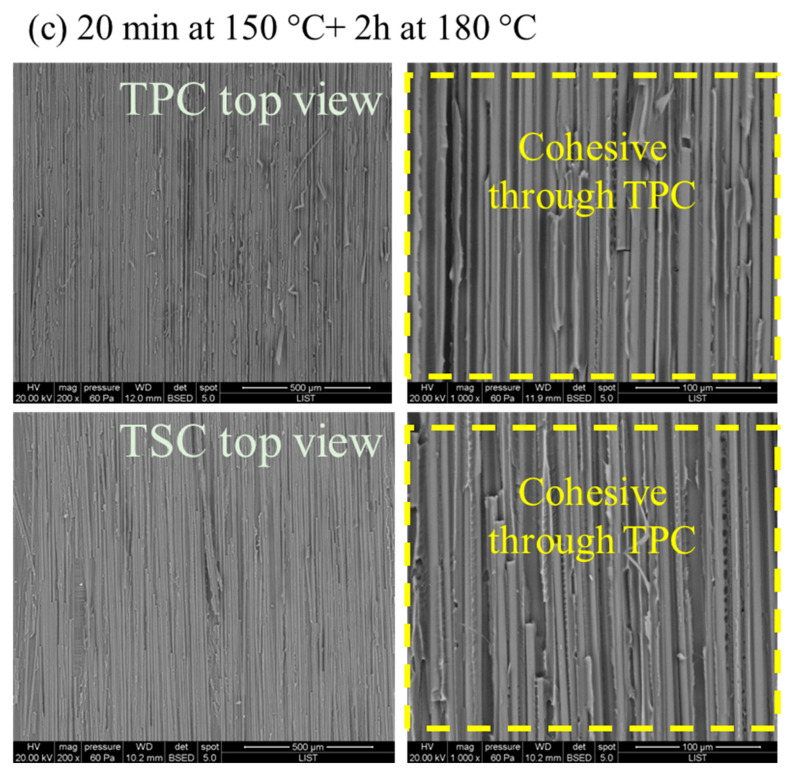
SEM images of the fracture surfaces of the co-cured samples after peel tests: (**a**) 2 h at 180 °C, (**b**) 2 h at 140 °C + 2 h at 180 °C, (**c**) 20 min at 150 °C + 2 h at 180 °C.

To further explore the microscopic assessment of cohesive strength at the interface, Figure 4 showcases a spectrum of failure modes, ranging from adhesive and cohesive failures within the co-cured interface to the potential failure of the thermoplastic tape. The observed failure mode varies depending on the specific dwell cure cycles employed. For the single dwell cure cycle (Figure 4a, 2 h at 180 °C), we identified a partial cohesive failure through the thermoplastic composite (TPC), distinguished by a notably rough surface. Additionally, as seen in Figure 4a, adhesive failure was observed, characterized by a smooth surface. One explanation for the adhesive failure is the highly crosslinked epoxy, which obstructed the formation of a complete interface. In the case of two dwell cure cycles, it is apparent that the surface was rougher compared to that of a single dwell cure cycle (Figure 4b,c). Nevertheless, adhesive failure on the TPC side was noticeable when curing at 140 °C for 2 h followed by curing at 180 °C for 2 h. Conversely, for the scenario involving curing at 150 °C for 20 min and then 2 h at 180 °C, the dominant mechanism was cohesive failure through TPC. Nonetheless, as depicted in Figure 3b, a sudden increase or decrease in peel force was evident, leading to the dissipation of release energy caused by the presence of micro-cracks during the peeling process. Consequently, despite cohesive failure, there was a lower level of reproducibility observed among the samples.

**Figure 5:**
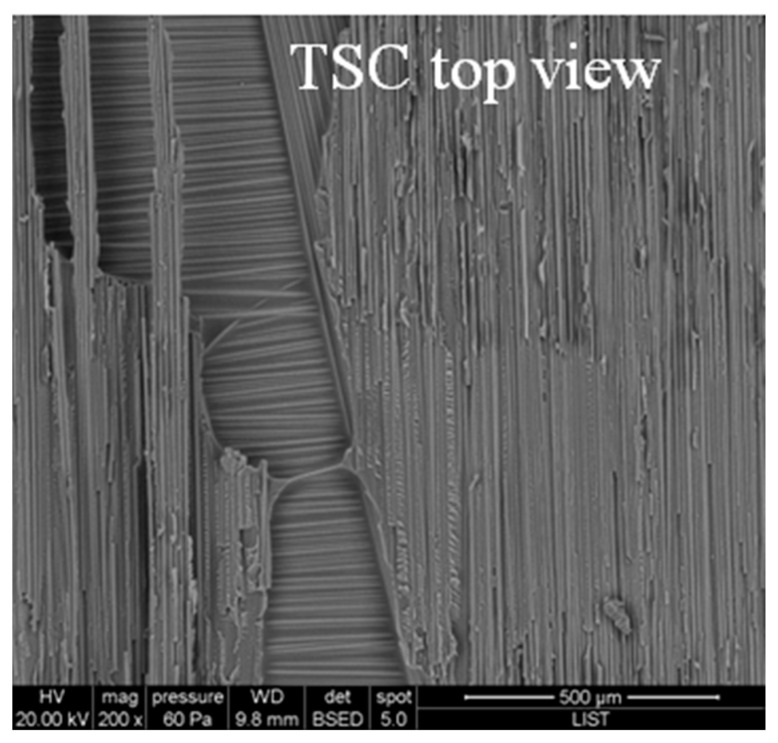
SEM images of the fracture surfaces of the co-cured samples at 150 °C for 2 h and at 180 °C for 2 h. TPS stands for thermoset.

For the co-cured joint interface subjected to a curing cycle of 2 h at 150 °C followed by 2 h at 180 °C, the surface of the fracture exhibited greater roughness compared to the other samples cured under different cycles. Generally, a rougher fracture surface indicates increased material toughness. As depicted in Figure 5, we observe ‘tied’ fibres in this scenario, where strips of the matrix have been cleanly peeled from the fibre surface. These occurrences result from the fibres being pulled from the surfaces during the peeling test, consequently inducing localised shear at the interface. These localised deformations are well-correlated with changes in crack speed (stick–slip growth) or the partial arrest of the 10^−3^ crack front.

These findings offer exciting prospects for the assembly of hybrid structures through co-curing. However, the underlying physical mechanisms responsible for submicron interdiffusion need further exploration. The interdiffusion of resin molecules into thermoplastic materials is acknowledged to bring about alterations in the microstructure through processes like dissolution and swelling. This, in turn, leads to modifications in the physical and mechanical properties of the thermoplastics, influencing diffusion and interphase formation [50]. However, a comprehensive understanding of the interdiffusion process involving a reactive resin into thermoplastics during the co-curing process remains largely unexplored due to several challenges and complexities. These challenges include: (i) the continual changes in the molecular weight and structure of the thermosetting resin during the curing process; (ii) the substantial influence of curing reactions and interdiffusion on environmental conditions; and (iii) the unclear mechanism of diffusion and post-diffusion phenomena, such as gelation and phase separation [51]. Our ongoing experimental investigation of the interphase involves reproducing the diffusion phenomena without carbon fibre. This approach allows for more precise sample preparation conducive to sub-micron observations using atomic force microscopy (AFM).

## 4. Conclusions

This study introduces an innovative technique involving the co-cure bonding of dissimilar carbon fibre/epoxy (CF/Ep) and carbon poly-ether-ether-ketone (CF/PEEK) composites. An enhancement in mechanical interlocking was achieved by subjecting the C/PEEK tape surface to atmospheric plasma treatment. Key aspects of this approach include: (i) the implementation of a one-step co-cure bonding process that preserves the integrity of the thermoset components; and (ii) the simplicity of this welding process, obviating the need for an intermediary thermoplastic interlayer. The insights derived from this research showed that:

(1) The curing cycle exerts influential contributions to thebonding characterisation of co-cured interfaces of PEEK/RTM6. The results showed that curing temperature and time are of high importance, and hence the peeling test results do help in understanding which cure cycles can lead to strong bonds between PEEK/RTM6.
(2) While peeling force for the cure cycle of 2 h at 140 °C + 2 h at 180 °C showed higher values in some of the iterations, the curing cycle of 2 h at 150 °C + 2 h at 180 °C showed reproducible results, and the adhesion between PEEK and RTM6 tends to be more stable.

Further study would have to be made to measure the impact of peeling velocities where stick–slip behaviour becomes prominent. In forthcoming investigations, we plan to explore how surface roughness affects the specific characteristics of the stick–slip pattern.

## Figures and Tables

**Table 1:** Nominal specification of CF/PEEK tape: *T_g_* is glass transition temperature [28,29,30,34].

Commercial Name	Characteristics					
	Thickness (µm)	Fibre Fraction Volume (%)	*T_g_* (°C)	Recrystallisation Temperature (°C)	Crystallinity (%)	Manufacturer
Aptiv^®^2000	25	54	143	160	8	Victrex^®^

**Table 2:** Nominal specification of RTM6-2 [31,32,35].

Commercial Name	Characteristics				
	Recommended Cure Cycle	*T_g_* (°C)	Gel Time at 140 °C (min)	Resin Injection Temperature (°C)	Manufacturer
HexFlow^®^ RTM6-2	120 min at 180 °C	High	95	80 under vacuum/ low pressure	Hexcel^®^

**Table 3:** Nominal specification of twill-woven carbon fabric for manufacturing of a composite laminate.

Style	Material	Number of Fibre	Weave Pattern	Areal Density
HexTow AS4C	Carbon fibre	3K	2 × 2 twill	200 g/m^2^

**Table 4:** Statistical analysis of maximum peel force of six iterations for each corresponding curing cycle.

Curing Cycle	Mean of Maximum Peel Forces (M) (N/mm)	Standard Deviation (sd)	Coefficient of Variation (sd/M × 100)
2 h at 140 °C + 2 h at 180 °C	2.10	0.5	21.9
20 min at 150 °C + 2 h at 180 °C	1.71	0.45	25.4
2 h at 150 °C + 2 h at 180 °C	1.37	0.2	15.2
2 h at 150 °C	1.17	0.11	9.7
2 h at 180 °C	1.22	0.071	5.7

## Data Availability

Data are contained within the article.
